# Promoting constructive feedback on preprints with the FAST principles

**DOI:** 10.7554/eLife.78424

**Published:** 2022-04-27

**Authors:** Sandra Franco Iborra, Jessica Polka, Iratxe Puebla

**Affiliations:** 1 ASAPbio San Francisco United States

**Keywords:** Point of view, preprints, peer review, peer review of preprints, FAST principles

## Abstract

Ensuring that public feedback on preprints is focused, appropriate, specific and transparent (or FAST) will help to develop a thriving culture for reviewing and commenting on preprints.

Preprints are liberating. In the traditional publishing model, the gap between the submission of a manuscript to a journal and its publication after peer review is measured in months, sometimes years. With preprints, this gap comes down to days. This early and public availability of articles creates opportunities for other researchers to read, cite, and build on the work. It also enables others to respond to the work on social media or via a comment on the preprint platform. Examples include articles highlighting preprints (such as preLights), commentary on preprints from journal clubs (such as the cross-institutional journal club run by Mount Sinai, University of Oxford and Karolinska Institute), journals reviewing preprints (such as *eLife*), and new services for reviewing preprints (such as Review Commons and Rapid Reviews COVID-19).

In practice, most feedback on preprints is quite positive: indeed, one study of comments on preprints found that praise was more common than criticism by a factor of almost 2.5 (Malički et al., 2021). However, while we all like to receive positive feedback, there is also a valuable role for critical feedback: for example, debates and dialog between researchers with opposing models and interpretations can help to move a field of research forward. Such debates can also help students and other newcomers understand the open questions and frontiers of the field. Critical feedback, if framed constructively and delivered appropriately, can make research more robust.

While it could be argued that anyone with feedback on a preprint could just email the authors, the advantage of public feedback is that everyone can benefit from it and potentially join the conversation. Moreover, public feedback can help prevent the spread of misinformation and disinformation, as happened with a number of questionable preprints about COVID-19 (Oransky and Marcus, 2020; Reardon, 2021).

Many of us are accustomed to getting private feedback from reviewers on papers and grant applications, and this feedback can sometimes be unvarnished or even harsh, but it rarely sees the light of day. Public criticism, however, is much more uncomfortable, but there can also be a silver lining. Recently, for example, one of us (JP) posted a preprint (about preprints) on bioRxiv (Brierley et al., 2021), and a colleague left a comment suggesting we had drawn a spurious conclusion. From our point of view, we had been careful to avoid drawing that precise conclusion, and it was initially jarring to see this raised publicly. However, we realized that if this colleague felt that we had reached this conclusion, so might other readers, so we revised the preprint in order to bring more clarity to our argument. Importantly, the fact that this concern was raised publicly allowed us to reply publicly, to clear the air, and to address the issue in a way that was more visible to others who might have shared the concern. Ultimately, we now view this public criticism as a blessing in disguise.

So how do we move forward to a culture of constructive public dialog on preprints? First, we need to address some general problems with the way feedback is delivered in science, be it in public or private: as David Drubin once wrote in an editorial in *Molecular Biology of the Cell*: “Any jackass can trash a manuscript, but it takes good scholarship to create one” (Drubin, 2011). Second, because public feedback on preprints is truly public (everyone can provide feedback, and everyone is able to read it) we need to look beyond the reviewer role and discuss cultural norms applicable to the broader research community and also the public. The FAST principles were developed to meet these challenges.

## FAST: Focused, Appropriate, Specific and Transparent

**Figure 1.**
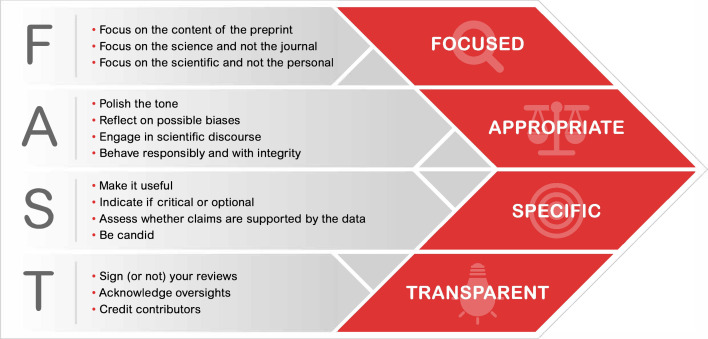
The FAST principles for providing public feedback on preprints. The 14 FAST principles are clustered around four broad themes: focused, appropriate, specific and transparent. See Franco Iborra et al., 2022 for more details.

If we want to promote public feedback on preprints, we need to articulate the behaviors that reflect the constructive and inclusive interactions we would like to see. With that goal, we convened a Working Group that included researchers, editors, preprint review platforms and funders to discuss two questions: what practices enable the benefits of public feedback on preprints? What are the cultural barriers that prevent us from embracing public preprint review, and how do we overcome these barriers? The output of the Working Group are the FAST principles, a set of 14 principles clustered around four broad themes (Franco Iborra et al., 2022; Figure 1):

- Focused: on the science and the scope of the manuscript.
- Appropriate: reflect on your tone and motivation; we encourage all actors to behave with responsibility and integrity as with any form of scientific exchange.
- Specific: clear, actionable and precise feedback that is useful for the authors to improve their work and distinguishes between critical versus minor issues.
- Transparent: public feedback allows for increased transparency, allowing reviewers to disclose their identity and/or expertise and an open forum to credit all contributors.

The FAST principles include existing advice on how to engage with journal peer review, but they also go further to reflect the public nature of comments on preprints, the possibility to comment without an editor, and the involvement of a wider community. We envision the community as an important stakeholder with the ability to engage and interact with the comments provided by authors and reviewers. Of course, just like authors and reviewers, the community has a responsibility to ensure that all exchanges remain constructive, inclusive and positive.

Different organizations can make use of the FAST principles in different ways. The platforms that host commentary and reviews on preprints can incorporate the principles into their framework for contributors, and use them as part of any moderation processes they have in place for posted reviews.

While most journals already have their own resources for authors and reviewers, editors may find that the FAST principles contain useful elements (such as, for example, guidance for authors on how to respond to comments from reviewers). For journals seeking to bring feedback on preprints into their editorial process (O’Grady, 2021), the principles provide a framework for selecting preprint reviews that are suitable for the journal’s editorial process. In addition, for journals that post reviews alongside preprints, such as *eLife* and *EMBO Journal* (eLife, 2021; Pulverer and Lemberger, 2019), the principles can guide what elements of the review are most relevant to post publicly with the preprint.

Institutions seeking to recognize peer review activities, including the peer review of preprints, in staff performance reviews could signal that for reviews of preprints to be eligible for recognition, they must have been completed in line with the principles.

And most importantly, we hope that the FAST principles are also a valuable resource for researchers. Early-career researchers seeking to build review skills, including students participating in courses on peer review, could be encouraged to use the principles when writing their reviews (as in the course Biochemistry 200A at the University of California San Francisco), as could participants in preprint journal clubs.

Of course, we do not expect the culture of public commenting on preprints to change overnight. Most of those who have previously participated in peer review have only done so in the context of a closed process run by a journal, where their comments were only seen by the editor and authors, and where most often comments were not signed. Moving the conversation into the public space means being much more proactive about how we develop our review, what biases we may have, and what choices of language we make to prevent misinterpretation. It may mean putting in that bit of extra effort when drafting our comments, but given the overall benefits of public feedback on preprints, we view this as a worthwhile investment.

## Looking forward

New platforms and communities are developing around the review of preprints, and it is likely that new approaches and tools will allow us to react to and comment on preprints in ways that are not possible today. The norms around public commentary on preprints are still fluid, but it is important to set the foundations for a constructive and inclusive culture at this early stage. The FAST principles outline behaviors that will encourage and lead to such a culture. And as activities around preprint feedback grow in visibility, scope and variety, we expect that standards will also evolve, and we look forward to seeing the principles develop to meet the changing needs of the community.

Preprints have brought researchers new freedom for how and when they communicate their research. By enabling new ways of public interaction, preprints also allow researchers to make new choices about how they engage in feedback and scientific discourse. In the context of a preprint, feedback is focused on the science, and not on the requirements of a particular journal. This gives an individual the opportunity to comment on a specific aspect of a preprint, or work in a group to provide a collective review. Importantly, by encouraging this public interaction and articulating what we value in preprint feedback, we can also enable new ways to reward reviews as scholarly contributions in their own right.

Journal peer review is valued by the scientific community, but it is seldom a conversation. Scientists value interactions with their peers about their work, and they gather feedback and ideas from face-to-face conversations, email enquiries, discussions at conferences and, sometimes, social media. The spirit of the FAST principles is to open a new avenue for this communication through preprint feedback. An added benefit is that it will give both the broader scientific community and the general public a chance to participate. We invite all members of the scientific community to be part of this conversation.
